# Myiasis and Its Relation to Pellagra

**Published:** 1918-09

**Authors:** Edward F. Wright

**Affiliations:** Greenville, Texas


### Myiasis and Its Relation to Pellagra.

EDWARD F. WRIGHT, M. D., GREENVILLE, TEXAS.

The presence of the larvae of flies in the gastro-intestinal tract has been considered of such rare occurence that our text book gives but very little attention to it; but from recent observation I have been led to believe that it is of much more frequent occurrence than we suppose and is responsible for gastro-intestinal and systemic symptoms that bear a very strong resemblance to those of pellagra.

I have within the past twelve months had occasion to observe and treat nine cases of intestinal myiasis; five of which were pellegrins, one an epileptic, and three that showed no other symptoms but acute gastro-intestinal irritation.

The first case I found by accident while making an examination of the feces for hookworm. I found the stool almost alive with maggots. This man developed all the symptoms of pellagra during the time I was trying to find something to rid him of the larvae. After a thorough cleaning with any purgative his condition would improve but in a very short time he would be as bad as ever. He was eventually freed of the larvae by the use of coal-oil in salol-coated capsules given every four hours during the day.

About the time that the above mentioned case got well I found one of two years standing who has been treated for pellagra and for the myiasis. She gave a history of having passed larvae ntermittingly for two years regardless of season and she knew well when to expect the passage of the larva by the appearanc of a marked distention of the ascending colon accompanied by a marked depression, abdominal pain, sore mouth, gastric irritation and diarrhea, accompanied by discharges of the larvae in large numbers. She took a trip to west Texas and stayed several months during which time she passed the larvae as at home. In this case I took some of the larvae and hatched them out into the fly and sent same to Washington for examination and classification and they were classed as Sarcophagii. I gave this case the coal oil treatment and she has been free for about six months and all her symptoms of pallagra has disappeared.

I was lead to believe from the history of the above case that the larvae became the fly in the cacum and that the fly bred the larvae and kept up a viscious circle otherwise I could not account for the presence of the larvae in the winter and summer seasons and for the further fact that she passed them on her visit to West Texas.

I confirmed the above assumption in the epileptic in that he passed the dead flies after taking the coal oil.

I had occasion to treat two cases of pellegra in children whose mother had died of the disease some few years before and I found both of these children and an older member of the family suffering from myiasis. I used the ordinary treatment for pellagra and the old grandmother beat me to the coal oil treatment when she discovered the condition by exhibiting a decoction of Jerusalem Oak in no uncertain dossage and seemed to get as permanent results as I did with the coal oil. The symptoms of pellagra disappeared under the combined treatment.

I do not wish to convey the impression that I believe that myiasis is the cause of pellagra but I do think that it gives rise to symptoms bearing a strong resemblance to those of pellagra and that a small per cent. of the cases of pellagra that get well in a short time and are easily handled are due to the presence in the colon of larvae of the blow fly and I further believe that when the eggs of the fly are taken into the stomach they find lodgment in the ceacum and develop in the larvae and then into the fly and that the fly thrives and reaches adult life in the ceacum and breeds and produces the eggs from which the larvae are hatched, for in the above mentioned cases I eliminated every possible source of infection through the food by an appeal to the sufferers and they were sufficiently intelligent and concerned to give earnest cooperation and yet the larvae appeared in the stools in a short time after the use of thymol and santonic in large doses often repeated.

Since writing the above I have had but limited opportunity for observing cases of pellagra on account of illness, but so far I have not seen a case of pellagra that did not pass these maggots. I saw a case of marked pellagra in March of this year and she was passing the worms and her improvement was rapid after treatment directed towards the removal of the worms.

I have had reported to me by Dr. Scott of Royse three cases of myiasis in one family and one of these is a pellagrin. Dr. Wright of Farmersville has reported several cases of pellagra that came under his observation that were passing the maggots in large quantities, and I am persuaded to believe that there is a very close relation between myiasis and pellagra and I believe that should the doctors have their pellagra patients observe the action of the bowels they will find that myiasis complicates, at least, if it does not cause, a large per cent of the cases, and that upon close observation many cases of acute digestive disturbances will be found due to the presence of the larvae of the Sarcophagii.

